# The Role of Lipid Raft Aggregation in the Infection of Type II Pneumocytes by *Mycobacterium tuberculosis*

**DOI:** 10.1371/journal.pone.0045028

**Published:** 2012-09-14

**Authors:** Kari Fine-Coulson, Barbara J. Reaves, Russell K. Karls, Frederick D. Quinn

**Affiliations:** Department of Infectious Diseases, University of Georgia, Athens, Georgia, United States of America; Institut de Pharmacologie et de Biologie Structurale, France

## Abstract

Dynamic, cholesterol-dense regions of the plasma membrane, known as lipid rafts (LR), have been observed to develop during and may be directly involved in infection of host cells by various pathogens. This study focuses on LR aggregation induced in alveolar epithelial cells during infection with *Mycobacterium tuberculosis* (*Mtb*) bacilli. We report dose- and time-dependent increases in LR aggregation after infection with three different strains at multiplicities of infection of 1, 10 and 100 from 2–24 hr post infection (hpi). Specific strain-dependent variations were noted among H37Rv, HN878 and CDC1551 with H37Rv producing the most significant increase from 15 aggregates per cell (APC) to 27 APC at MOI 100 during the 24 hour infection period. Treatment of epithelial cells with Culture Filtrate Protein, Total Lipids and gamma-irradiated whole cells from each strain failed to induce the level of LR aggregation observed during infection with any of the live strains. However, filtered supernatants from infected epithelial cells did produce comparable LR aggregation, suggesting a secreted mycobacterial product produced during infection of host cells is responsible for LR aggregation. Disruption of lipid raft formation prior to infection indicates that *Mtb* bacilli utilize LR aggregates for internalization and survival in epithelial cells. Treatment of host cells with the LR-disruption agent Filipin III produced a nearly 22% reduction in viable bacteria for strains H37Rv and HN878, and a 7% reduction for strain CDC1551 after 6 hpi. This study provides evidence for significant mycobacterial-induced changes in the plasma membrane of alveolar epithelial cells and that *Mtb* strains vary in their ability to facilitate aggregation and utilization of LR.

## Introduction

*Mycobacterium tuberculosis* (*Mtb*), the causative agent of tuberculosis (TB), has infected an estimated one-third of the world’s population with potentially 60–90% of these individuals harboring the latent form of the disease [1]. While significant research has been focused on *Mtb*/host interactions, there remains a paucity of data defining attachment and internalization of bacilli with the epithelial cells that line the alveolus. *Mtb* bacilli have been shown to infect and replicate in alveolar epithelial cells both in vitro and in vivo [2]–[6]. It is believed that these interactions play an important role during both early and chronic infection by influencing the host immune response to the pathogen [7]–[9]. Therefore, it is imperative to better define the events that occur during *Mtb* infection of the alveolar epithelial cell.

Over the last 15–20 years our understanding of eukaryotic cell membrane organization has changed dramatically. Areas of the plasma membrane, known as lipid rafts (LR), have been described as dynamic regions within the membrane enriched in cholesterol, glycosphingolipids, sphingomyelin, phospholipids with acyl chains, glycosylphosphatidylinositol (GPI)-linked proteins as well as other membrane proteins such as innate immune receptors [10]–[12]. Studies utilizing phototonic force microscopy and fluorescent resonance energy transfer have established the size of rafts in an unperturbed cell system to be approximately 5 nm-50 nm in diameter which would be undetectable by light microscopy [12]–[14]. However, other studies have demonstrated that stimuli applied to the plasma membrane, such as bacteria and/or the toxins they produce, can induce aggregation of LR to a size observable by confocal microscopy [15]–[20].

Lipid raft aggregation on target host cells in response to interactions with infectious agents has yielded interesting results for viruses and bacteria alike. Influenza virus has been shown to associate with LR via hemagglutinin and neuraminidase, and examination of enveloped virions post-budding shows a significant number of raft domains within the viral envelope [21]. Further it has been demonstrated that LR are important for HIV-1 viral budding [22]. During bacterial infections, LR have been shown to induce important changes in lipid raft formation. Some bacterial proteins, produced during infection, help facilitate hijacking of the host cell. Binding of cholera toxin subunit B to ganglioside (GM1) found in LR is required for uptake of the toxin [23]. Further, various bacterial proteins have been shown to induce LR aggregation to promote host cell responses to the pathogen. For example, treatment of macrophages with Listeriolysin-O (LLO) has been shown to induce large “super” aggregates of LR which facilitate signaling through receptor tyrosine kinase domains, suggesting LR aggregation may facilitate an innate immune response during infections with *Listeria monocytogenes* [15].

LR have also been shown to promote internalization of various bacterial pathogens. This function appears to be linked to caveolin proteins found within the microdomains. Caveolin proteins associate intimately with LR forming invaginations known as caveolae. These invaginations have been shown to facilitate the uptake and colonization of pathogenic strains of *Escherichia coli*, *Salmonella typhimurium* and *Pseudomonas aeruginosa* [24]–[26]. Caveolin-1-deficient mice have been shown to be more resistant to pulmonary *P. aeruginosa* infection; this correlates with LR/caveolae-dependent endocytosis of the bacteria in type I alveolar epithelial cells [27]–[29]. Further, these studies also found a signaling function for LR platforms in the same host cells in response to *P. aeruginosa* attachment. Collectively, this work demonstrates that LR serve important functions during bacterial invasion.

Previous studies have also investigated the role of LR and cholesterol aggregation during *Mtb* infection of the macrophages and mast cells. Shin *et al*. demonstrated translocation of Toll-like Receptor-2 (TLR-2) to LR in macrophages treated with the *Mtb* 19k Da lipoprotein LpqH [30]. Gatfield and Pieters (2000) performed staining with the LR-disruption agent Filipin to demonstrate cholesterol clustering around *Mycobacterium bovis* BCG during infection and that subsequent depletion of cholesterol inhibited uptake of the bacilli in macrophages [31]. Interestingly, *Mtb* entry into mast cells has also been shown to be LR dependent [32]. Other studies have demonstrated that mycobacterial cell wall lipids such as lipoarabinomannan can become incorporated into membrane rafts found in phagosomes to inhibit phagosome/lysosome fusion in macrophages [33], [34].

To date, no investigation has been conducted to characterize variations in LR aggregation in non-phagocytic cells during infection. Further, no studies have compared the variance in plasma membrane response to multiple strains of *Mtb* and the subsequent role of the aggregates produced. To evaluate the role of LR aggregation in *Mtb* pathogenesis within alveolar epithelial cells, type II pneumocytes were infected with virulent laboratory strain H37Rv as well as strains, CDC1551 and HN878 [35]–[37]. The objective of this study was to examine dose- and time-dependent LR aggregation in response to different *Mtb* strains in the alveolar type II epithelial cell.

## Results

### Live *Mycobacterium tuberculosis* Strains Induce Dose and Time-dependent Lipid Raft Aggregation in A549 Cells

**Figure 1:**
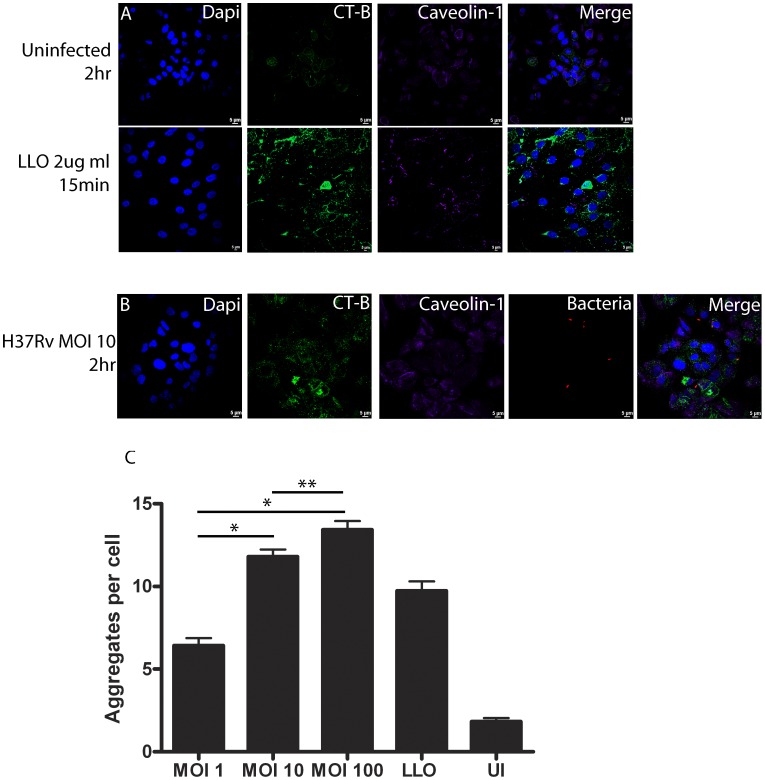
*Mtb* bacilli induce LR aggregation at 2 hr post infection. A549 type II alveolar epithelial cells were infected with *Mtb* strain H37Rv at three bacterial multiplicities of infection (MOI) and analyzed at 2 hr post infection (hpi) by confocal microscopy as described. Uninfected and Listeriolysin-O (LLO) treated cells were also analyzed as negative and positive controls for lipid raft (LR) aggregation, respectively. Images demonstrate that infection with H37Rv (MOI 10) produced an increase in the number of LR aggregates compared to uninfected controls (A–B). Quantification of LR aggregates produced for MOI 1, 10 and 100 demonstrate LR aggregation comparable to LLO positive controls (p-value >0.05) (C). Significantly more LR aggregates per cell (APC) were detected from H37Rv infected cells compared to uninfected controls (p-value <0.001). A dose-dependent increase in LR aggregation was also noted among H37Rv infections MOI 1 (∼6 APC), MOI 10 (∼12 APC), and MOI 100 (∼14 APC) (^*^p-value <0.001; ^**^p-value <0.05). Infections were performed in duplicate and experiments repeated three times. A total of 15 fields were imaged per coverslip, incorporating approximately 30–50 host cells per field. Images were captured at 63x magnification and LR aggregation assessed using ImageJ software. Results represent the average of three experiments.

Previously published work has established that *Mycobacterium tuberculosis* (*Mtb*) bacilli attach and become internalized by type II alveolar epithelial cells between 2–6 hr post infection (hpi) [3], [6]. Thus, A549 cells were infected with *Mtb* strain H37Rv, a virulent laboratory strain, and examined at 2 hpi to determine if live *Mtb* bacilli were capable of inducing lipid raft (LR) aggregation during initial contact with the host cell. Three different bacterial multiplicities of infection (MOI) were employed to determine if induction of LR aggregation was dose-dependent. Listerolysin-O (LLO), produced by *L. monocytogenes*, has been shown to induce LR “super” aggregation and was thus applied to uninfected cells as a positive control for these studies [15]. Aggregation was assessed by quantifying the colocalization of Cholera Toxin-B (CT-B)-stained puncta and caveolin-1 antibody labeling. An example of how these aggregates were identified is shown in Fig. S3. At 2 hpi, confocal images show numerous LR aggregates in both LLO treated and H37Rv infected A549 cells (MOI = 10) compared to uninfected controls (Fig. 1 panels A & B). Further, quantification of these images demonstrates a dose-dependent increase in the number of LR aggregates from bacterial multiplicity of infection (MOI) 1 (∼6 aggregates per cell) to 100 (∼14 aggregates per cell) (Fig. 1 panel C; p-value <0.001). Infection with H37Rv at each MOI produced significantly more LR aggregates per cell compared to uninfected controls (p-value <0.001). These data indicate that live *Mtb* bacilli are capable of inducing significant LR aggregation in type II alveolar epithelial cells upon initial contact.

**Figure 2:**
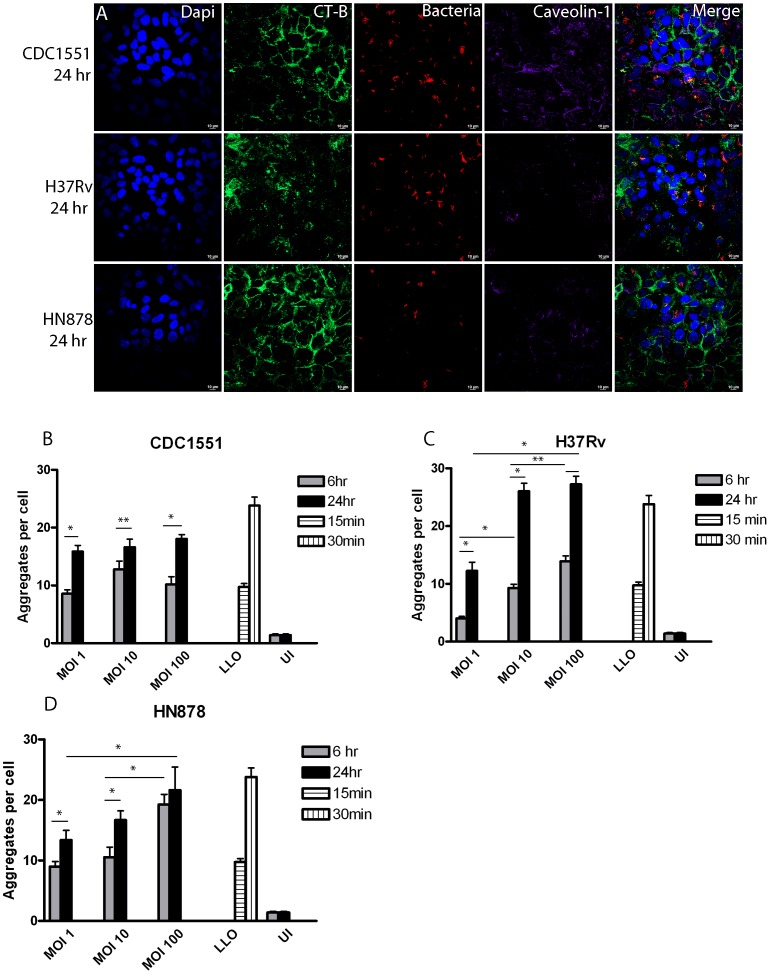
*Mtb* strains induce time- and dose–dependent increase in LR aggregation. A549 type II alveolar epithelial cells were infected at three MOI’s, 1 (Fig. S1 A), 10 (A) and 100 (Fig. S1 B) and specimens prepared for confocal microscopy as described. A549 cells treated with Listeriolysin-O (LLO) as positive controls were assessed 15 and 30 min post treatment (data not shown) Images show that infections with strain CDC1551, HN878 and H37Rv produce substantial LR aggregation at 6 hpi (data not shown) and 24 hpi (A). Quantification of microscopic images at 6 and 24 hpi demonstrated a time-dependent increase in LR aggregation for CDC1551 (B), H37Rv (C) and HN878 (D ) (*p-value <0.001 and ^**^<0.05). Dose-dependent increases in LR aggregation were also significant for H37Rv and HN878 at 6 and 24 hpi (*p-value <0.001 and **p-value <0.05). At MOI 100, strains HN878 and H37Rv consistently produced higher levels of LR aggregation compared to CDC1551. Infections were performed in triplicate and experiments repeated three times. A total of 15 fields were imaged per coverslip, incorporating approximately 30–50 host cells per field. Images were captured at 63x magnification and LR aggregation assessed using ImageJ software. Results represent the average of three experiments.

Infection of A549 cells with *Mtb* strain H37Rv induced LR aggregation, thus to investigate if multiple strains of *Mtb* were capable of inducing LR aggregation, monolayers of A549 cells were infected with *Mtb* strains HN878, CDC1551 and H37Rv. LR aggregates were evaluated by confocal microscopy at 6 and 24 hpi, when *Mtb* bacilli have been shown to be internalized and establish stable infection in A549 cells [6]. At a MOI of 10, confocal images at 24 hpi demonstrate substantial LR aggregation for all three strains (Fig. 2 panel A) Images suggest some strain related differences in the number of LR aggregates at 24 hpi. HN878 and H37Rv appear to produce higher levels of LR aggregation compared to CDC1551 (Fig. 2 panel A). Similar observations were made for MOI 1 and 100 at both time points (Fig. S1 & S2 panels A–B).

LR aggregates produced within A549 cell membranes after infection with H37Rv, HN878 and CDC1551 were quantified from confocal images for each MOI at 6 and 24 hpi using colocalization of CT-B and caveolin-1 as the marker for LR aggregation. A time dependent increase in LR aggregation was noted for all three strains (Fig. 2 panels B, C & D). For example, at an MOI of 10 the number of LR aggregates increased from ∼10 to ∼27, at 6 and 24 hr, respectively, during infection with H37Rv (Fig. 2 C). Significant dose dependent increases in the number of LR aggregates were also observed for H37Rv and HN878 at 6 and 24 hpi compared to uninfected controls. As visual assessment of the confocal images suggested, some differences in the number of LR aggregates was observed between strains. Infections with strain CDC1551 produced the lowest overall number of LR aggregates of the three *Mtb* strains with ∼17–20 aggregates per cell observed at 24 hpi (Fig. 2 panel B). The level of aggregation observed at 6 and 24 hr was comparable to LLO positive controls for all three strains. These data suggest that *Mtb* bacilli induce LR aggregation in a dose and time dependent manner and that individual isolates vary in their ability to induce LR aggregation.

### *Mycobacterium tuberculosis*-derived Total Lipids and Culture Filtrate Proteins Induce Limited Lipid Raft Aggregation

**Figure 3:**
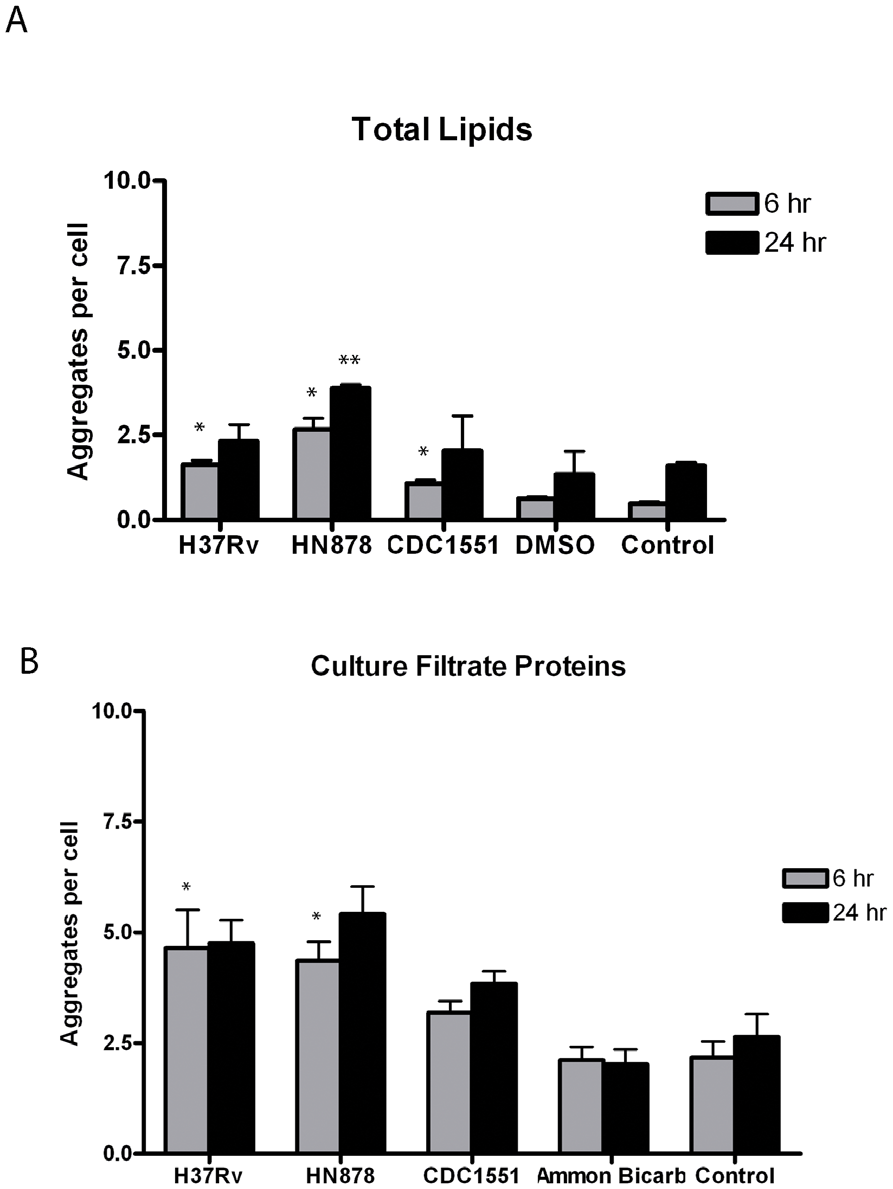
Treatment of A549 cells with total lipids or culture filtrate proteins from *Mtb* strains produces limited LR aggregation. A549 cells treated with total lipids (TL) or culture filtrate proteins (CFP) from all three strains were processed for confocal microscopy at 6 and 24 hr post-treatment as described. At 6 hr post-treatment, a significant increase in LR aggregation was observed in TL treated A549 cells compared to uninfected controls (*p-value <0.05) (A). At 24 hr post-treatment HN878 TL produced significantly higher aggregate numbers compared to uninfected controls (**p-value <0.001) (A). It should be noted that LR aggregation for HN878, CDC1551 and H37Rv at 24 hr post-treatment was not significantly different from uninfected and DMSO controls (p-value 0.065, 0.977 and 0.737, respectively) (A). Compared to controls, treatment with CFP from CDC1551 did not produce statistically different LR aggregation values at 6 and 24 hr post-treatment (p-value = 0.598 and 0.379, respectively) (B). LR aggregation for HN878 and H37Rv was significantly different from controls at 6 hr post-treatment (*p-value = 0.033 and 0.012, respectively) (B). Experiments were performed in triplicate and repeated three times. A total of 15 fields were imaged per coverslip, incorporating approximately 30–50 host cells per field. Data presented are the average of three experiments. Images to obtain LR data were captured at 63x magnification.

The novel observation of LR aggregation in A549 cells in response to infection with *Mtb* bacilli was further examined to determine if constitutively expressed mycobacterial components were capable of inducing the LR aggregation observed during infection with live bacteria. To answer this question, total lipid extracts (TL) and culture filtrate proteins (CFP), proteins secreted by the bacteria when grown in broth culture, were obtained for each strain and applied to alveolar epithelial cells. After treatment and labeling for LR, images were quantified for induction of aggregates as described (see above and Fig. S3). Significantly fewer LR aggregates were observed for all three strains with TL treatment compared to live bacterial infections at 6 and 24 hpi (Fig. 2 panels B-D & Fig. 3 panel A). Total lipid extracts from all three strains induced <4 aggregates per cell at both time points. Interestingly, at 6 hpi, HN878 TL produced increased numbers of aggregates (2.8) compared to H37Rv (1.8) and CDC15551 (1.1), similar to the trend observed with live infections at the same time point. This pattern was repeated at 24 hpi though no significant differences were noted among strains. However, the number of LR aggregates produced by TL treatment was significantly decreased compared to aggregation induced by live bacterial infection with each strain (p-value <0.001).

Epithelial cell monolayers treated with CFP from each strain were processed for confocal microscopy and LR aggregates quantified. The number of aggregates observed for H37Rv and HN878 CFP was significantly increased compared to controls (Fig. 3 panel B). There was no significant difference noted in the number of LR aggregates between 6 and 24 hr post treatment. Overall, CFP treatments produced significantly fewer aggregates compared to infections with live bacteria for all three strains at 6 and 24 hpi (p-value <0.001).

Collectively, these data suggest that the mycobacterial components, TL and CFP from broth-grown cultures, do not demonstrate the degree of LR aggregation observed during infection with live *Mtb* strains.

### Gamma-Irradiated Bacteria Induce Limited Lipid Raft Aggregation

**Figure 4:**
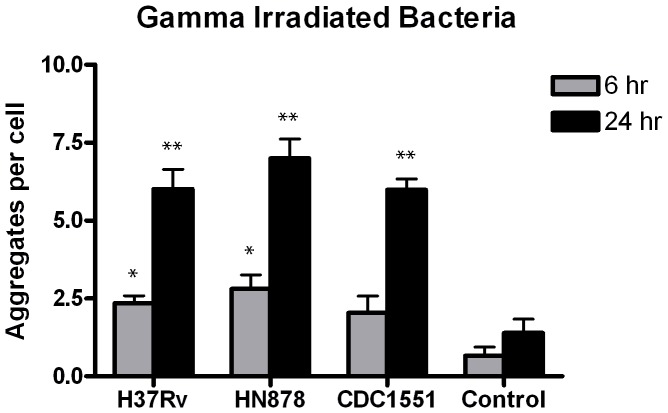
Addition of gamma-irradiated *Mtb* strains produces low level A549 cell LR aggregation. Gamma-irradiated CDC1551, HN878 and H37Rv bacilli were added to monolayers at an inoculum of 100 bacilli per host cell. The cells were then processed for confocal microscopy and LR aggregation defined by CT-B staining and quantified as described. Controls without bacilli produced LR aggregation at 6 and 24 hr post-treatment comparable to controls for other previous experiments. At 6 hr post-treatment a significant increase in LR aggregation was observed with strains H37Rv and HN878 compared to controls (*p-value <0.001). LR aggregation with all three strains at 24 hr post-treatment was significantly increased compared to controls (**p-value <0.001). Experiments were performed in triplicate and repeated three times. A total of 15 fields were imaged per coverslip, incorporating approximately 30–50 host cells per field. Data presented are the average of three experiments. Images were captured at 63x magnification.

To determine if the binding of inactivated but intact *Mtb* bacilli to the A549 cell membrane was sufficient to induce the level of LR aggregation observed during infection with live mycobacterial strains, aliquots of gamma-irradiated bacilli were obtained for all three strains and applied to epithelial cell monolayers as described. We chose to use GI bacteria rather than heat killed or alcohol killed bacteria as they are more likely to retain key morphological and chemical features that may play a role in LR aggregation [38], [39]. While differences in LR aggregation between strains at both time points was not significant, addition of irradiated HN878 bacilli did produce a slight increase in LR aggregate number compared to the other two strains which might be attributed to differences in cell wall components observed in previous studies (Fig. 4) [40]. Strain CDC1551 induced aggregation similar to H37Rv at 6 and 24 hpi (Fig. 4). An increase in total LR aggregates was noted from 6 to 24 hpi for each strain in a manner similar to infection with live strains. However, the number of LR aggregates observed overall was significantly decreased compared to live infections.

**Figure 5:**
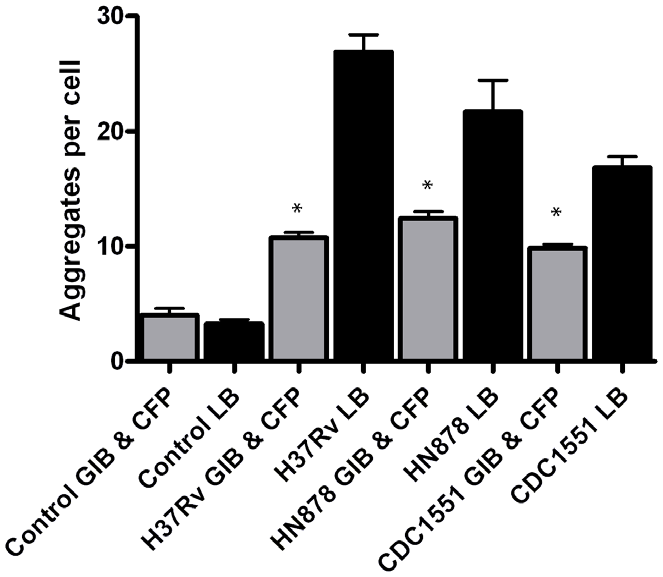
LR aggregation produced by gamma-irradiated *Mtb* strains and CFP does not equal aggregation observed with live bacteria. Data from figures 3 and 4 were combined for comparison with LR aggregation data from live bacteria experiments. Less than 55% of the aggregation observed with live *Mtb* infection is detected when CFP or gamma-irradiated strains H37Rv, HN878 and CDC1551 are used (*p-value <0.001, 0.01 and 0.05, respectively). (GIB- gamma irradiated bacteria; LB- live bacteria; CFP- culture filtrate proteins).

To test whether accumulated input from gamma-irradiated bacilli and CFP would result in the same induction of LR observed with live strains, summation of these experiments was performed for comparison. The number of LR aggregates induced by gamma-irradiated bacilli and CFP is indicative of some basal level of aggregation induced at 6 and 24 hpi. However, these components only account for 40–55% of the LR aggregation seen at 24 hpi with live H37Rv, HN878 and CDC1551 (p-value <0.001, 0.01 and 0.05 respectively) (Fig. 5). These data suggest that products secreted by live bacteria during an infection are responsible for the level of LR aggregation observed.

### Mycobacterial-proteins Produced during Infection Induce Lipid Raft Aggregation

**Figure 6:**
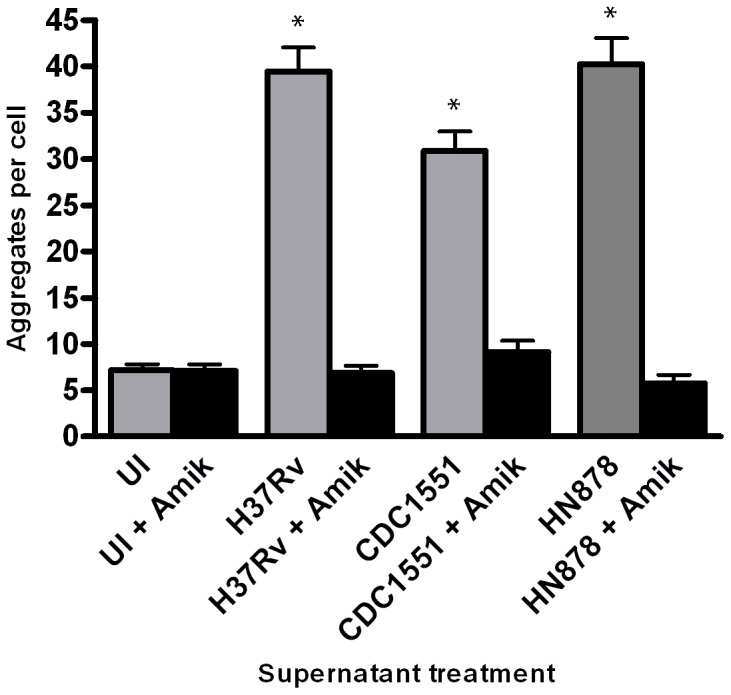
Proteins secreted by live *Mtb* bacilli induce LR aggregation in alveolar epithelial cells. A549 cells were infected with CDC1551, HN878 and H37Rv that were either pretreated with amikacin or untreated at MOI 100 for 24 hr. Supernatants were then micro-filtered and applied to fresh monolayers of A549 cells seeded onto coverslips and allowed to incubate for 24 hr. Coverslips were prepared for confocal microscopy as described. Supernatants from infections with untreated bacteria produced significantly more LR aggregation compared to supernatants from infections with amikacin-treated bacteria (*p-value <0.001). Infections and subsequent supernatant treatments were performed in duplicate and repeated twice. A total of 15 fields were imaged per coverslip, incorporating approximately 30–50 host cells per field. Images to analyze LR aggregation were captured at 63x magnification. (UI = uninfected cells; Amik = supernatants from bacteria pretreated with Amikacin).

To determine if the LR-inducing product(s) from live bacilli is a secreted protein expressed during infection of host cells, monolayers of epithelial cells were infected with *Mtb* strains that were pre-treated with amikacin, to inhibit active bacterial protein synthesis, or infected with non-treated bacilli. After 24 hpi, supernatants from the infected cells and controls were collected then filtered to remove bacteria. The filtered supernatants were then applied to a fresh monolayer of A549 cells and incubated for 24 hr. Cells were fixed and prepared for confocal analysis as described. No significant difference in the number of LR aggregates was noted between control supernatants, uninfected and uninfected with amikacin, or supernatants from A549 cells infected with amikacin-treated bacteria (p value >0.05) (Fig. 6). However, A549 cells treated with filtered supernatants from infections with strains not treated with antibiotic produced a significant increase in LR aggregates compared to amikacin-treated controls (p-value <0.001). Supernatants from H37Rv and HN878 infections produced ∼37 and ∼38 aggregates per cell, respectively, compared to ∼5 and ∼4 aggregates per cell induced by supernatants from the same mycobacterial strains pretreated with amikacin. A similar trend was observed with supernatants from CDC1551 infections (∼30 aggregates per cell) compared to supernatants from amikacin-treated bacteria (∼7 aggregates per cell). In these experiments, a slight increase in total LR aggregate numbers was observed compared to live bacteria experiments although not statistically significant (p-value >0.05). These data confirm that mycobacteria-induced LR aggregation results from proteins secreted by live *Mtb* strains during infection of epithelial cells.

### Toll-Like Receptors do not co-localize with Mycobacteria-induced Lipid Raft Aggregates

**Figure 7:**
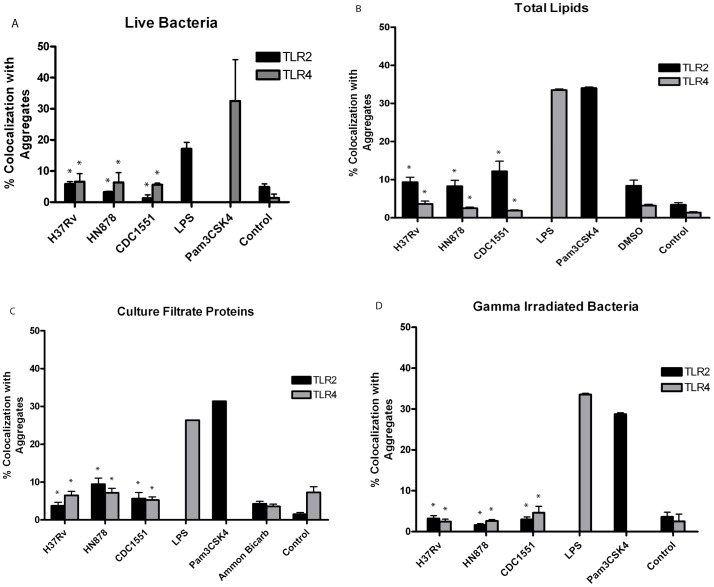
TLR-2 and TLR-4 do not colocalize with LR aggregates after infection/treatment with *Mtb* bacilli or cell fractions. A549 cells were infected with live *Mtb* strains CDC1551, HN878 or H37Rv at MOI 100 or treated with total lipids (TL), culture filtrate proteins (CFP) and gamma-irradiated cells for 24 hr, as described. Colocalization of anti-TLR-2 and anti-TLR-4 antibodies with LR aggregates was analyzed from confocal images using Manders coefficient values. Infections with CDC1551, HN878 and H37Rv produced significantly less TLR-2 and TLR-4 colocalization compared to positive controls (*p-value <0.001) (A). There were no significant differences between TL-treated cells and DMSO controls at this time point (B) (TLR-4, p-value = 0.532 to 0.983; TLR-2, p-value = 0.775 to 1.0). TLR-2 and TLR-4 colocalization for controls and CFP-treated cells was comparable (C). At 24 hr post-TL (B) and –CFP (C) treatment, colocalization of receptors was significantly decreased compared to positive controls (*p-value <0.001). These findings also held true for epithelial cells infected with gamma-irradiated bacteria from all three strains (*p-value <0.001) (D). Images were collected at 63x magnification and analyzed using ImageJ JACoP plugin. Infections were performed in duplicate and repeated three times.

It was previously demonstrated during infection with various pathogens that aggregation of LR serves to protect monocytes and epithelial cells through increased innate immune receptor signaling [11], [41]. To determine if the *Mtb*-induced LR aggregation served as platforms for host receptors, Toll-Like Receptor (TLR) 2 and 4, both important cellular receptors which initiate innate responses to mycobacteria, were examined for colocalization with CT-B stained puncta [30], [42], [43]. Positive controls PamCSK4 and LPS were used for TLR-2 and TLR-4 translocation to LR, respectively. Epithelial cells infected with live *Mtb* strains produced <6% colocalization of TLR-2 antibodies with CT-B stained aggregates compared to 32% with positive controls (Fig. 7, A). Likewise, live infections produced <7% colocalization compared to 15% with LPS (Fig. 7, A). In both experiments TLR colocalization with LR aggregates was significantly lower than colocalization with positive controls (p-value <0.001). These data suggest that LR aggregates are not functioning as platforms for TLR 2 or TLR 4 in response to live *Mtb* infection.

To determine if basal LR aggregation observed within A549 cell membranes after addition of CFP, TL and gamma-irradiated-whole bacteria may serve to produce the low level of TLR colocalization demonstrated with live bacteria, epithelial cells were treated with these components as described and evaluated for colocalization. Treatment with TL, CFP and gamma-irradiated bacteria from all three *Mtb* strains produced <10% colocalization with TLR-2 and <12% colocalization with TLR-4 compared to approximately 30% colocalization with positive controls (Fig. 7, B-D). Collectively, these data suggest that constitutively-expressed components may be responsible for low level TLR colocalization with LR aggregates and subsequently some signaling functions. However, the degree of aggregation observed with live *Mtb* strains compared to TLR-2 and TLR-4 colocalization suggests alternative functions for these platforms.

### *Mycobacterium tuberculosis* Bacilli Colocalize with and Utilize Lipid Raft Aggregates for Internalization

**Figure 8:**
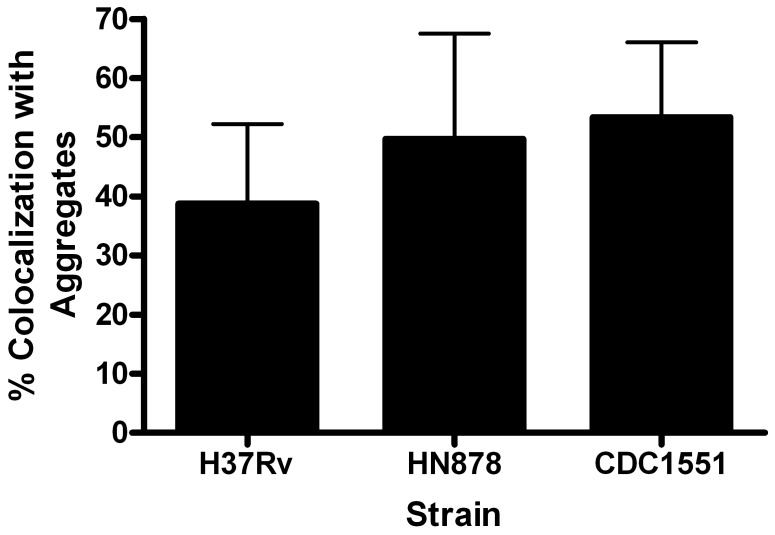
CDC1551, HN878 and H37Rv bacilli colocalize with LR aggregates. A549 cells were infected with live *Mtb* strains CDC1551, HN878 and H37Rv, and colocalization of fluorescent bacteria with LR aggregates was quantified as described. No statistical difference was noted for LR colocalization between strains at 24 hpi. Infections were performed in triplicate and repeated three times. Analysis was performed on 15 fields per coverslip using ImageJ JACoP plugin. Images were captured at 63x magnification.

Previous studies demonstrated the important association of plasma membrane cholesterol with the entry of *Mtb* bacilli in macrophages [31]. To determine if the observed LR aggregation serves to pool plasma membrane cholesterol and facilitate mycobacterial entry into epithelial cells, bacilli colocalizing with CT-B/caveolin-1 puncta were visually quantified. At 24 hpi, 40–52% of H37Rv, HN878 and CDC1551 bacilli viewed per field colocalized with CT-B puncta (Fig. 8). These data suggest that live bacilli associate with approximately half of the LR aggregation observed and that these areas may help facilitate bacterial entry into the host cell.

**Figure 9:**
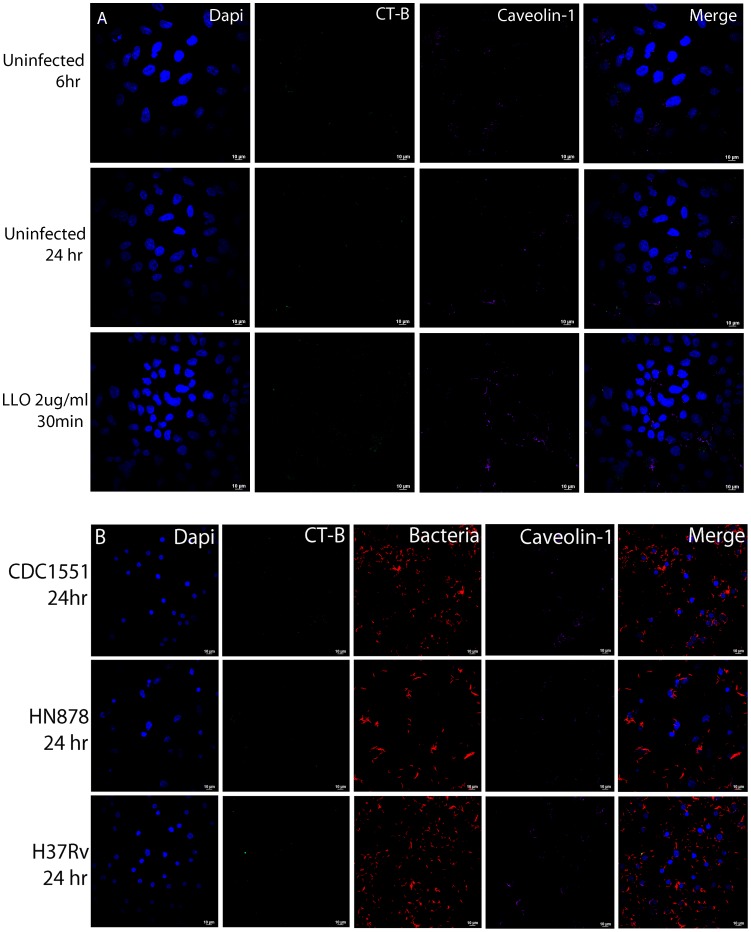
Treatment of epithelial cells with Filipin III disrupts mycobacterial-induced LR aggregation. A549 cells were treated with cholesterol-binding, LR-disruption agent Filipin III and LR aggregation observed for controls and infections with all three live *Mtb* strains. Confocal microscopy demonstrated an absence of CT-B puncta at 6 and 24 hpi for controls (A) and live *Mtb* strains (B). Images were collected at 63x magnification. Infections were performed in triplicate and repeated three times.

Disruption of the LR entity using this sterol binding agent has been shown to eliminate caveolae-dependent endocytosis [44]. To evaluate if LR aggregate formation induced by *Mtb*-bacilli was crucial for internalization, epithelial cells were treated with the LR/cholesterol disrupting agent Filipin III and viable numbers of intracellular bacteria were quantified. Confocal images verified disruption of LR aggregates at 6 hpi (data not shown) and 24 hpi with controls and all three strains of *Mtb* (Fig. 9, panel A & B). To confirm that Filipin treatment did not disrupt overall internalization mechanisms control experiments were conducted with fluorescent dextran. A549 cells treated with Filipin for 6 hr showed no significant decrease in endocytosis of dextran (data not shown).

**Figure 10:**
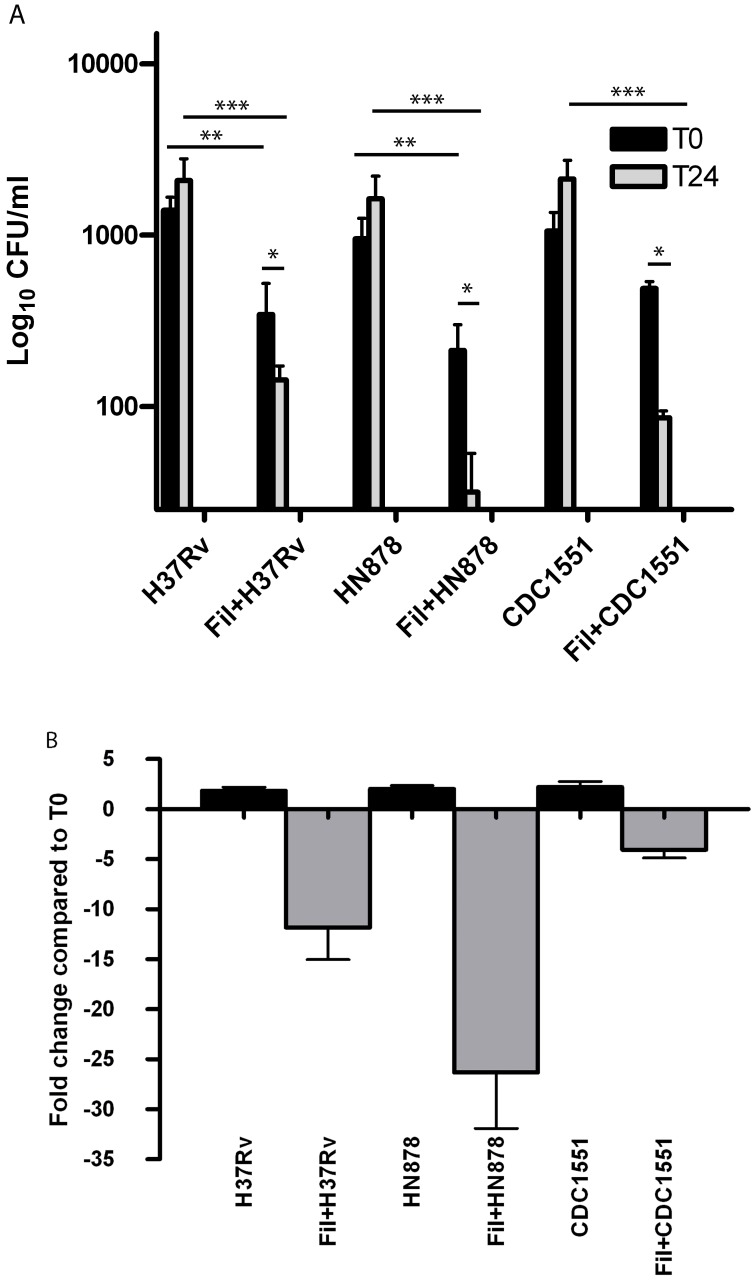
Filipin-treated A549 cells have significantly fewer viable intracellular bacteria after uptake. Filipin- and non-Filipin treated epithelial cells were infected with CDC1551, HN878 and H37Rv at MOI 100 as described. Intracellular bacterial viability over time (amikacin protection assays) was quantified by lysing host cells and plating on Middlebrook 7H10 medium at T0, after 6 hr of bacterial uptake, and 24 hpi (T24)(A). Numbers of viable bacteria were significantly decreased in Filipin-treated compared to non-treated host cells infected with HN878 and H37Rv at T0 (**p-value <0.001; <0.01, respectively) and T24 (***p-value <0.001) (A). No significant difference was found between drug-treated and non-drug treated host cells infected with CDC1551 at T0 (p-value >0.05). A significant difference was found between treatments for CDC1551 infections at T24 (***p-value <0.01). The calculated fold change from T0 to T24 indicates a significant decrease in the number of viable intracellular bacteria over time (B) (*p-value <0.001). Infections were performed in triplicate and repeated three times.

In parallel, Filipin- and non-Filipin-treated host cells were infected with all three *Mtb* strains, and viable count experiments conducted as described. After 6 hr of bacterial uptake, at T0 infections with HN878 and H37Rv in Filipin-treated A549 cells saw a significant reduction in intracellular bacteria compared to non-drug treated host cells (p-value <0.001; <0.01 respectively) (Fig. 10, panel A). While not statistically significant, a 7.5% reduction in intracellular bacterial numbers was observed for CDC1551 between non-treated and Filipin-treated A549 cells at the same time point (Fig. 10, panel A). These data suggest that disruption of LR aggregation negatively impacts internalization of *Mtb* bacilli in epithelial cells and that the kinetics and mechanism of internalization appear to vary for different *Mtb* strains.

Comparison of numbers of viable bacteria from T0 to T24 was also performed to determine if lack of LR-mediated internalization impacted survivability of *Mtb* bacilli once inside the host cell. Filipin-treated cells infected with H37Rv saw a 12–fold reduction in numbers of viable bacteria from T-0 to T-24 (Fig. 10, panel B). Further, a 5-fold and 25-fold reduction was observed with CDC1551 and HN878 Filipin-treated infections, respectively (Fig. 10, panel B). These data suggest that inhibiting LR-mediated internalization of *Mtb* bacilli in epithelial cells does impact intracellular numbers of those bacteria.

## Discussion

Cell biologists have studied the dynamic changes that can occur in the plasma membrane of various mammalian cell types allowing for aggregation of lipid rafts (LR). These structures are thicker than adjoining regions of the membrane and rich in cholesterol, sphingolipids as well as transmembrane proteins. In an unperturbed environment, line tension, or boundary energy, prevents significant aggregation of the 10–200 nm-sized LR [45]. Various LR markers were employed in this study to characterize the presence, density and quantity of these structures in response to infection with *M. tuberculosis* (*Mtb*) bacilli. Cholera toxin- B (CT-B), which binds to ganglioside (GM1) found in lipid rafts (LR), has been utilized in numerous microscopy studies to examine the presence of LR on mammalian cell plasma membranes [11], [15], [46]–[48]. Caveolin proteins, which bind to cholesterol in LR, have also been used as a set of markers to identify these structures [26]. Caveolin-1 is one example which has been shown to coalesce to form flask-like invaginations called caveolae [49]–[51]. Caveolae LR are considered to be a subset of the LR population and thus it should be noted that quantification using caveolin-1/CT-B colocalization might under represent the total LR population present on the host cell plasma membrane [52], [53]. However other studies have utilized these markers to demonstrate LR involvement in normal cell functions and disease processes [54]–[58].

Aggregation of LR has been shown to occur in response to various stimuli. Cross-linking of transmembrane proteins found in LR has been shown to induce aggregation of these cholesterol dense areas [59], [60]. Studies employing bacterial proteins, such as listeriolysin O (LLO) have been shown to induce sizable LR clusters that can be viewed microscopically [15], [19], [61]. To determine if *M. tuberculosis* (*Mtb*) bacilli were capable of inducing lipid raft aggregation, A549 human type II alveolar epithelial cells were infected with three isolates of live *Mtb* bacilli at three different MOI. Time and dose-dependent increases in LR aggregation were observed from 2–24 hours post infection (hpi). It was also shown that total lipids (TL) and culture filtrate protein (CFP) components purified from broth-grown cultures, and gamma-irradiated bacilli did not produce the same degree of LR aggregation observed during infection with the actively-growing strains.

Subsequent studies were performed to examine LR aggregation facilitated by mycobacterial-proteins expressed and secreted specifically during infection with viable bacilli. Filtered supernatants from infected type II pneumocytes were able to induce a similar level of LR aggregation observed when live bacteria were present. Thus, in a manner similar to secreted LLO, *Mtb* proteins produced and secreted during infection with live bacilli are capable of inducing aggregation of cholesterol-dense LR. This unknown mycobacterial protein(s) could induce LR aggregation through binding of protein receptors found within the rafts, analogous to GM1 and cholera toxin. Future work will focus on identifying this protein(s) as well as the mechanism of LR aggregation.

Some significant differences in the number of LR aggregates were also noted between live *Mtb* strains at each time point. Using the same number of infecting bacteria, strain CDC1551 induced fewer aggregates per cell (APC) than H37Rv and HN878 at 6 and 24 hpi. Previously published observations of CDC1551 infection in animal models supports a phenotype of decreased virulence compared to HN878 [36], [37]. This virulence phenotype could be correlated with diminished LR aggregation observed in this study. CDC1551 has been shown to stimulate a robust Th1 immune response in aerosol-infected mice and guinea pigs and less host death compared to H37Rv infections [36]. Conversely, strain HN878 has been shown to stimulate a relatively lower Th1 response and faster time-to-death in these same animal models [36], [37], [62], [63]. The observed increased virulence associated with strain HN878, as quantified by the rapid time-to-death of infected animals has been attributed, in part, to unique phenolic glycolipids (PGL-tb) found in the cell wall of this strain [40], [64]. HN878 is a member of the *Mtb* Beijing/W genotype known to have an intact polyketide synthase gene (*pks1-15*) required for PGL-tb synthesis. These data could correlate with the increased level of LR aggregation noted after addition of the HN878 TL fraction. However, PGL-tb is likely not solely responsible for the enhanced virulence of HN878 since studies by Sinsimer *et al.* (2008) have shown that other factors produced by this strain can contribute to the development of this phenotype [64]. Our future studies will examine purified PGL-tb from strain HN878 to determine if this factor alone can significantly contribute to the increased LR aggregation observed.

Lipid rafts and toll-like receptors (TLR) have been shown to have important signaling functions during *Mtb* infection of macrophage cell lines [30]. To evaluate this in epithelial cells, A549 monolayers were treated with CFP and TL mycobacterial components, gamma-irradiated cells or infected with live bacilli from three *Mtb* strains; TLR-2 and TLR-4 colocalization with LR aggregates was evaluated. The data presented here indicated that unlike macrophages, LR aggregation does not function as a platform for TLR2 and TLR4 accumulation during *Mtb* infection of epithelial cells. It is possible that human carcinoma cell lines do not produce innate immune responses in a manner analogous to healthy human type II pneumocytes. However, studies with A549 cells have demonstrated the presence and up-regulation of TLR-2 and -4 responses to infection with *Klebsiella pneumoniae* [65]. Additionally, positive controls from this study demonstrated a significant increase in TLR colocalization with LR aggregates supporting the assertion that LR aggregates do not predominately serve as platforms for innate immune receptors during *Mtb* infection of epithelial cells.

*Chlamydia trachomatis*, uropathogenic *Escherichia coli*, *Listeria monocytogenes, Pseudomonas aeruginosa* and *Salmonella enterica* have been shown to utilize LR as a means of internalization in various cell types [28], [46], [66], [67]. To determine if the LR aggregates induced in our studies function as a means for internalization of *Mtb* bacilli in epithelial cells, colocalization of aggregates and bacteria was examined. For each *Mtb* strain, approximately half of the bacteria viewed in each microscopic field colocalized with LR puncta and caveolin-1 antibody. Invaginations formed in LR, caveolae, have been shown to internalize viruses as well as bacteria and their toxins in epithelial cells [22], [41]. We have demonstrated that disruption of LR aggregation using the cholesterol binding agent Filipin III significantly diminished the number of intracellular viable bacteria for all three strains after 6 hr of infection. The sterol-binding agent Filipin prevents aggregation of cholesterol which has been shown to be crucial for the stability of caveolae and thus use of this agent selectively inhibits caveolae-dependent endocytosis [44]. Other studies have shown that entry of *Mtb* bacilli into type II pneumocytes is dependent upon actin polymerization [3], [4]. Because actin polymerization contributes significantly to caveolae-mediated endocytosis, we hypothesize that LR/caveolae-dependent endocytosis may play an important role for entry of *Mtb* bacilli in non-phagocytic cells [68]. It should be noted, however, that even in the presence of Filipin, approximately 50% of the bacteria were able to gain entry to the host cell. These data support the assertion that *Mtb* bacilli are internalized in nonphagocytic cells through multiple mechanisms and thus future studies will focus on describing these events.

Studies examining *Mtb* and *Mycobacterium avium* infections in the macrophage have demonstrated that cholesterol aggregation in the plasma membrane is crucial for uptake and later recruitment of host proteins to the phagosomal membrane and prevention of lysosomal fusion [31], [33], [69]. Thus, the impact of Filipin pre-treatment on intracellular survival of *Mtb* bacilli was examined. A significant negative-fold change in the number of viable intracellular bacteria was seen for all three strains from T-0 to T-24 hpi in Filipin-treated host cells. This finding indicates that, similar to macrophages, *Mtb* bacilli require cholesterol aggregation for internalization and survival during infection of the epithelial cell. Cholesterol-dependent inhibition of lysosomal fusion may explain previous observations demonstrating the absence of lysosomal-associated markers with *Mtb*-containing endosomes in epithelial cells [6]. Future work will focus on investigating the contribution of cholesterol in the membrane of the bacteria-containing compartment to bacterial survival.

Aggregation of LR facilitated by *Mtb* infection contributes to bacterial internalization and survival in the alveolar epithelial cell. It will be important to identify and understand the function of the specific mycobacterial factors responsible for LR stimulation and if these may be related to virulence of different mycobacterial strains. It is also evident that *Mtb* bacilli interact with alveolar epithelial cells using mechanisms different from those observed with macrophages. Thus, these interactions between *Mtb* bacilli and epithelial cells deserve closer scrutiny in order to uncover the role these cells play in pulmonary disease.

## Methods

### Bacterial Culture

The following cultures were obtained through the NIH Biodefense and Emerging Infection Research Resources Repository, NIAID, NIH: *Mycobacterium tuberculosis* (*Mtb*), strains H37Rv (NR-13648), CDC1551 (NR-14825) and HN878 (NR-13647). *Mycobacterium tuberculosis* strains were grown in Middlebrook 7H9 broth supplemented with 0.5% glycerol, 0.05% Tween 80 and 10% ADC (strain CDC1551) or 10% OADC (strains HN878; H37Rv). For confocal microscopy, *Mtb* strains were transformed with plasmid pGCRED2 expressing DsRed2 and maintained by inclusion of hygromycin at 50 µg/ml. Plasmid pCGRed2 was a generous gift from Drs. Garry Coulson and Mary Hondalus, Department of Infectious Diseases, University of Georgia. Gamma irradiated bacteria also were obtained through NIH Biodefense and Emerging Infections Research Resources Repository, NIAID, NIH: *Mtb*, strains H37Rv (NR-14819), HN878 (NR-14821) and CDC1551 (NR-14820).

### Cell Culture

A549 human type II alveolar epithelial cells were obtained from ATCC (CCL-185) and maintained at 37°C, 5% CO_2_ in EMEM supplemented with 5% FBS. A549 cells were grown as monolayers to confluency, harvested with trypsin-treatment for 3 min at 37°C, and 5.0×10^5^ cells seeded onto sterile coverslips placed within 6-well Costar® dishes. The cells were allowed to adhere for 12 hr at 37°C in 5% CO_2_ and then infected with *Mtb* bacilli.

### Epithelial Infection

Epithelial cell monolayers were infected in 6-well dishes, as described, at the indicated MOI (1, 10, or 100) with the indicated *Mtb* strains. Bacteria were grown in 7H9 broth with gentle shaking to an OD_600_ 1.0 then centrifuged to remove broth media. The pellets were resuspended in EMEM supplemented with 5% FBS. To disperse innocula, bacteria vortexed for 5 min then passed through an insulin syringe and deposited directly into the appropriate tissue culture wells. This method of bacterial dispersion was confirmed by microscopy to produce single bacilli for infection (data not shown). Cold synchronization was performed to coordinate bacterial attachment. This procedure included incubation of the monolayer at 4°C for 2 hr; 1 hr preceding infection and 1 hr after addition of the bacteria. Subsequently, infected cells were incubated at 37°C in 5% CO_2_; this was considered time point 0. Experiments involving gamma irradiated bacteria were conducted in the same manner using an inoculum of 100 bacilli per host cell. Control experiments were conducted with Listeriolysin-O (LLO). Cells were treated with 2 µg/ml LLO (Diatheva s.r.l.) for 15 or 30 min at room temperature [15]. For Filipin studies, cells were pretreated and maintained throughout the infection with a final concentration of 5 µg/ml [70]–[72]. Filipin III was obtained from Sigma (F-4767) and reconstituted with DMSO. Control experiments with DMSO alone at the concentration applied in Filipin studies produced no cytotoxic effects. Host cells treated with Filipin were evaluated for cytotoxic effects microscopically and by the release of lactate dehydrogenase. Studies showed no deleterious effects produced by the drug at the dose utilized (data not shown). To verify that Filipin treatment did not significantly impact general endocytosis mechanisms, A549 cells were seeded onto coverslips in 6-well Costar® dishes. After 6 hr incubation with or without Filipin, 100 µg of 10,000 MW dextran-Texas Red (Invitrogen) was added to each well. Uptake proceeded at 37°C, 5% CO_2_ for 30 min after which cells were washed and fixed as described previously. An example of confocal images from these studies is presented in Fig. S4. Infections were performed in triplicate and experiments repeated three times.

For consistency, cold synchronization of A549 cells was employed prior to the addition of live or gamma irradiated bacteria and culture filtrate protein (CFP) or total lipid (TL) reagents (see below). A549 cells were incubated for 1 hr at 4°C before and after addition of cells, proteins or extracts. The host cells were then returned to 37°C (Time = 0 hr).

### Bacterial Culture Filtrate Protein and Total Lipid Treatment of Epithelial Cells

The following reagents were obtained through NIH Biodefense and Emerging Infection Research Resources Repository, NIAID, NIH: *Mtb* Culture Filtrate Proteins (CFP), strains H37Rv (NR-14825); HN878 (NR-14827); CDC1551 (NR-14826), and Total Lipids (TL), strains H37Rv (NR-14837); HN878 (NR-14839) and CDC1551 (NR-14838). For CFP experiments, monolayers of A549 cells were treated with 4 µg/ml of proteins based on previously published work [62], [73]. Control monolayers were treated with 0.01 M ammonium bicarbonate, the solution in which the culture filtrate proteins were dialyzed. Treatments were performed in triplicate and experiments repeated three times.

Total lipids were reconstituted in DMSO and monolayers of A549 cells treated with a final concentration of 2 µg/ml. Concentrations of TL used were based on previously published work and titrations performed prior to experimentation which microscopically assessed DMSO treatment and subsequent impact on host cell viability (data not shown) [35], [74], [75]. At the concentration applied, control monolayers of A549 cells treated with DMSO alone produced no adverse effects noted by microscopic examination (data not shown).

### Confocal and Immunofluorescence Microscopy

For confocal microscopy, A549 cells were grown as monolayers to confluency, harvested with trypsin-treatment for 3 min at 37°C, and 5.0×10^5^ cells seeded onto sterile coverslips placed within 6-well Costar® dishes. The cells were allowed to adhere for 12 hr at 37°C in 5% CO_2_ and then infected with *Mtb* bacilli or treated with gamma irradiated bacilli, CFP or TL as described. Specimens were fixed at indicated time points with 3.7% paraformaldehyde for 1 hr at 4°C. The specimens were washed 3 times with 1x PBS. Cells were permeabilized for 10 min with 0.1% Triton X-100. The samples were then blocked for 30 min with PBS containing 3% BSA. For lipid raft (LR) aggregation studies cells were incubated with rabbit polyclonal anti-caveolin-1 (Abcam) at 1∶200 dilution for 1 hr at room temperature (RT). The antibodies were detected using 1∶500 dilution goat-anti-rabbit 633 (Invitrogen) antibodies incubated for 1 hr at RT. Concurrent staining of detergent-resistant aggregates was performed using Cholera toxin B (CT-B) 488 (Invitrogen) at a dilution of 1∶200. Filipin-treatment experiments were conducted and labeled with CT-B and caveolin-1 as described. Images were obtained with either the Zeiss Axiovert 200 M and Apotome or a Nikon A1R confocal laser microscope system.

For Toll-like receptor experiments, A549 cells were seeded onto coverslips as described. Positive controls for TLR-2 stimulation were conducted using 1 µg/ml of Pam3CSK4 (InvivoGen) for 6 hr at 37°C. TLR-4 stimulation was performed using *E. coli* 0111:B4 strain LPS (InvivoGen) at 10 µg/ml for 6 hr at 37°C. Experimental wells of A549 cells were infected with *Mtb* strains CDC1551, HN878 and H37Rv at an MOI of 100. At 6 and 24 hpi specimens were fixed and prepared for labeling as described. Cells were incubated with either mouse monoclonal anti-TLR-4 (Santa Cruz) or mouse monoclonal anti-TLR-2 (Abcam) antibodies at 1∶200 dilution for 1 hr at RT. Both Primary antibodies were detected using goat-anti-mouse 633 antibodies (Invitrogen) at 1∶500 for 1 hr at RT. Separate experiments were conducted using mouse monoclonal transferrin receptor antibody (Zymed) at 1∶200 as a negative control for colocalization with LR. The antibody was detected using donkey-anti-mouse 633 (Invitrogen) at a dilution of 1∶500. CT-B 488 staining was also performed as described for each well during the secondary antibody incubation period. Images were obtained with a Nikon Eclipse TiE confocal microscope. All cells in each experiment were also stained with Dapi at 1∶500 for 1 hr at RT. All infections were performed in duplicate and experiments repeated three times.

### Supernatant Treatment

A549 cells were seeded onto 6-well plates as described and infected with *Mtb* strains CDC1551, HN878 and H37Rv. *Mycobacterium tuberculosis* strains were preincubated at 37°C for 1 hr in EMEM 5% FBS and 50 µg/ml amikacin to inhibit protein synthesis. Pretreated bacterial cells were applied to epithelial cell monolayers in a manner previously described in parallel with non-drug treated bacteria. Amikacin (50 µg/ml) was maintained on infected and control cells for the duration of the time course. Supernatants were pipetted into a 3 ml syringe and passed through Millex-GV 0.22 µm PVDF filters (Millipore) to remove bacteria. The filtered supernatants were applied to a new monolayer of A549 cells seeded onto coverslips and incubated at 37°C for 24 hr. Aliquots of filtered supernatants were applied to 7H11 agar plates supplemented with 0.5% glycerol, 0.05% T80, 10% ADC (CDC1551) or OADC (HN878; H37Rv) and 50 µg/ml hygromycin to verify removal of bacteria. Specimens were processed for confocal microscopy as described and imaged with a Zeiss Axiovert 200 M and Apotome. Infections were performed in duplicate and experiments repeated twice.

### Intracellular Bacterial Viability

Epithelial cell monolayers were seeded onto 24-well plates at 2.5×10^4^ cells per well and allowed to adhere for 12 hr prior to infection. Filipin- or non-Filipin-treated A549 cells were infected with *Mtb* strains at an MOI of 100 as described. After 6 hr at 37°C, medium was removed from each well, monolayers washed 3 times with 1x PBS and incubated for 2 hr in EMEM with amikacin (200 µg/ml), +/− Filipin and 5% FBS as described previously [6]. The medium again was removed, and monolayers washed with PBS and EMEM with amikacin, +/− Filipin and 5% FBS was applied; this was defined at time point 0 (T0). At T0 and T24, the cells were washed and lysed with 0.1% Triton X-100. Infections were performed in triplicate and experiments repeated three times.

### Assessment of Colocalization

Confocal images were obtained as described and imported into ImageJ 1.451/Java 1.60_20 software and analyzed using the JACoP plugin. LR aggregates were first quantified using ImageJ as described. Colocalization of TLR labeling with LR aggregates was evaluated in JACoP using Pearson and Manders coefficient. Bacteria colocalization with LR aggregates and/or caveolin-1 antibodies alone were analyzed in a similar manner.

### Quantification of LR Aggregates

Images of specimens were obtained as described and a total of 15 fields were imaged per coverslip for each experiment. Three coverslips were obtained for all three *Mtb* isolates, at each timepoint for all three experimental replicates (total = 54 coverslips per experiment). Once colocalization of CT-B and caveolin-1 was verified using Image J, JACoP plugin, aggregates were quantified and analyzed for size and area using ImageJ 1.451/Java 1.60_20 [76]. Numbers of aggregates were normalized to the number of host cells imaged per field to acquire an average number of aggregates per host cell/per field for each treatment condition.

### Statistical Analysis

Statistical significance of aggregate numbers and viable bacterial counts was examined by ANOVA and Tukey’s HSD post-hoc comparison (α = 0.05) using SPSS 17.0® statistical software.

## Supporting Information

Figure S1 **Infection with** ***Mtb*** **strains (MOI = 1) induce LR aggregation.** A549 cells were infected with H37Rv, HN878 and CDC1551 (MOI = 1) and incubated at 37°C, 5% CO_2_ for 6 hr (A) or 24 hr (B) as described. An increase in CT-B/caveolin-1 puncta is observed at 24 hpi (Quantification in Fig 2). Images were obtained at 63x magnification with a Nikon A1R confocal system equipped with a Nikon Eclipse TiE confocal microscope. Infections were performed in triplicate and repeated three times. Fifteen fields were imaged per coverslip for each experiment.
(TIF)

Figure S2 **Infection with** ***Mtb*** **strains (MOI = 100) induce LR aggregation.** A549 cells were infected with H37Rv, HN878 and CDC1551 (MOI = 100) and incubated at 37°C, 5% CO_2_ for 6 hr (A) or 24 hr (B) as described. An increase in CT-B/caveolin-1 puncta is observed at 24 hpi (Quantification in Fig 2). Images were obtained at 63x magnification with a Nikon A1R confocal system with a Nikon Eclipse TiE confocal microscopy. Infections were performed in triplicate and repeated three times. Fifteen fields were imaged per coverslip for each experiment.
(TIF)

Figure S3 **A magnified confocal image of A549 cells infected with CDC1551 demonstrates colocalization of LR markers.** A549 Type II alveolar epithelial cells were infected with CDC1551 (MOI = 10) and prepared for confocal microscopy as described. Images were obtained at 63x magnification with a Nikon A1R confocal system with a Nikon Eclipse TiE confocal microscope (A). The circle indicates the area from which the magnified image was obtained. The encircled area has been cropped and magnified to illustrate the colocalization of CT-B, Caveolin-1 and bacteria for the purposes of quantification as described in the methods (B).
(TIF)

Figure S4 **Treatment of A549 cells with Filipin III does not impact endocytosis of dextran.** Filipin- (A) or non-Filipin (B) treated A549 cells were incubated with 10,000 MW dextran-TR for 30 min and uptake assessed by confocal microscopy. Quantification using ImageJ indicated no significant difference in dextran endocytosed (data not shown). Dextran treatments were performed in duplicate and repeated twice.
(TIF)
