# Supplementary figures and images for: The Role of Lipid Raft Aggregation in the Infection of Type II Pneumocytes by Mycobacterium tuberculosis
Source: PLoS One. 2012 Sep 14;7(9):e45028. doi: 10.1371/journal.pone.0045028 (PMC3443240; doi:10.1371/journal.pone.0045028)

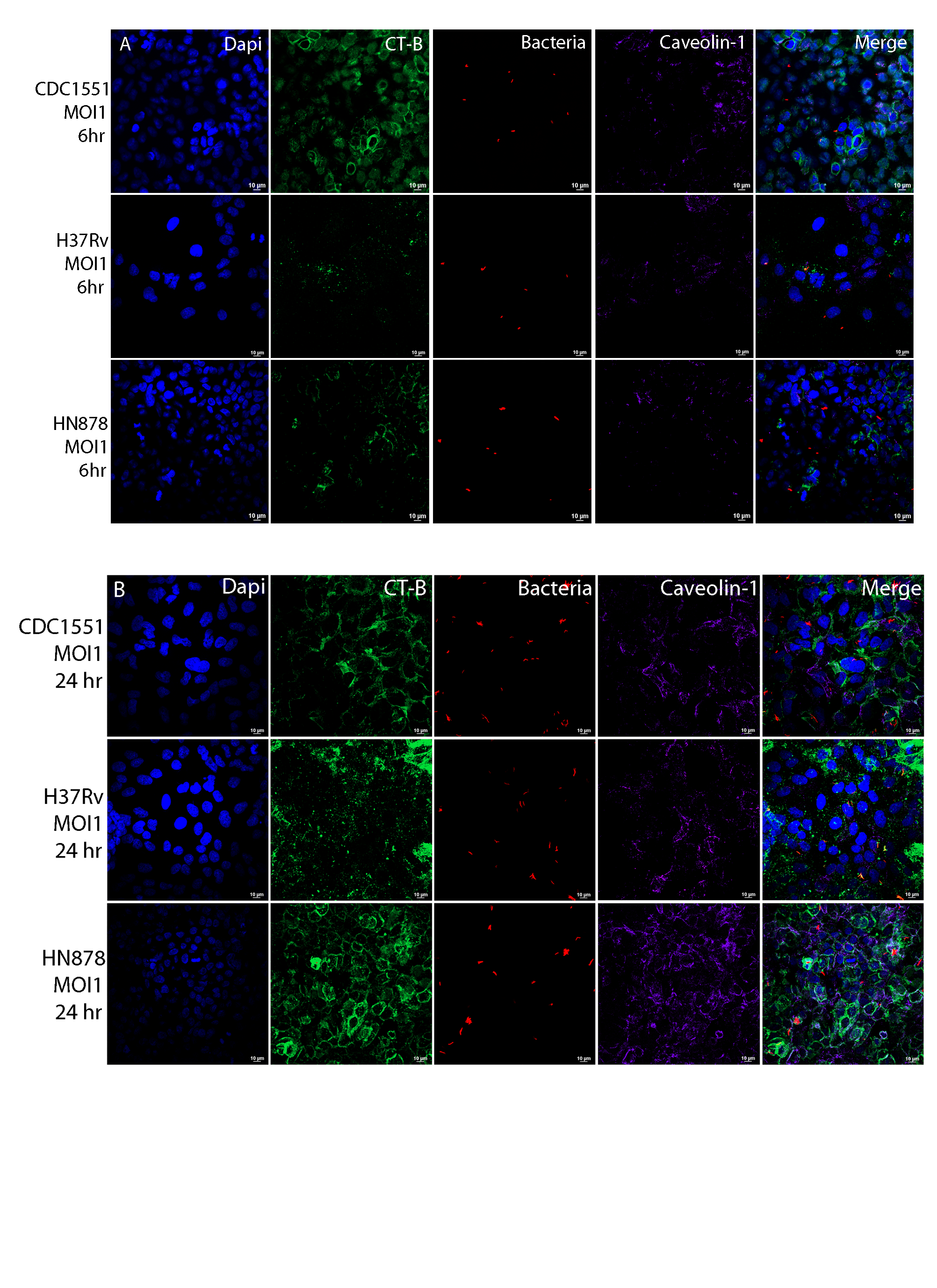

Supplement: Figure S1 — Infection with Mtb strains (MOI = 1) induce LR aggregation. A549 cells were infected with H37Rv, HN878 and CDC1551 (MOI = 1) and incubated at 37°C, 5% CO2 for 6 hr (A) or 24 hr (B) as described. An increase in CT-B/caveolin-1 puncta is observed at 24 hpi (Quantification in Fig 2). Images were obtained at 63x magnification with a Nikon A1R confocal system equipped with a Nikon Eclipse TiE confocal microscope. Infections were performed in triplicate and repeated three times. Fifteen fields were imaged per coverslip for each experiment. (TIF) [file pone.0045028.s001.tif]

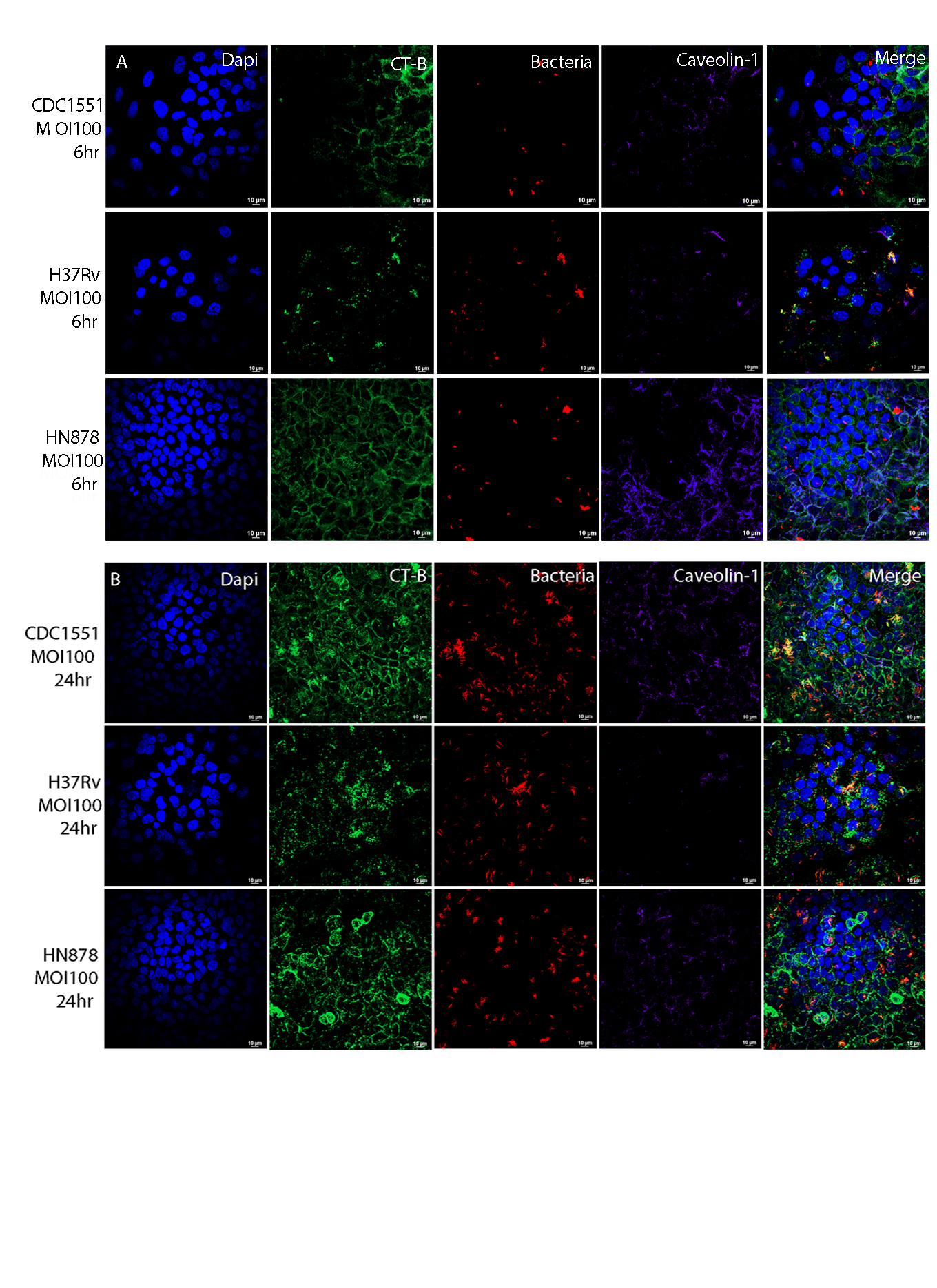

Supplement: Figure S2 — Infection with Mtb strains (MOI = 100) induce LR aggregation. A549 cells were infected with H37Rv, HN878 and CDC1551 (MOI = 100) and incubated at 37°C, 5% CO2 for 6 hr (A) or 24 hr (B) as described. An increase in CT-B/caveolin-1 puncta is observed at 24 hpi (Quantification in Fig 2). Images were obtained at 63x magnification with a Nikon A1R confocal system with a Nikon Eclipse TiE confocal microscopy. Infections were performed in triplicate and repeated three times. Fifteen fields were imaged per coverslip for each experiment. (TIF) [file pone.0045028.s002.tif]

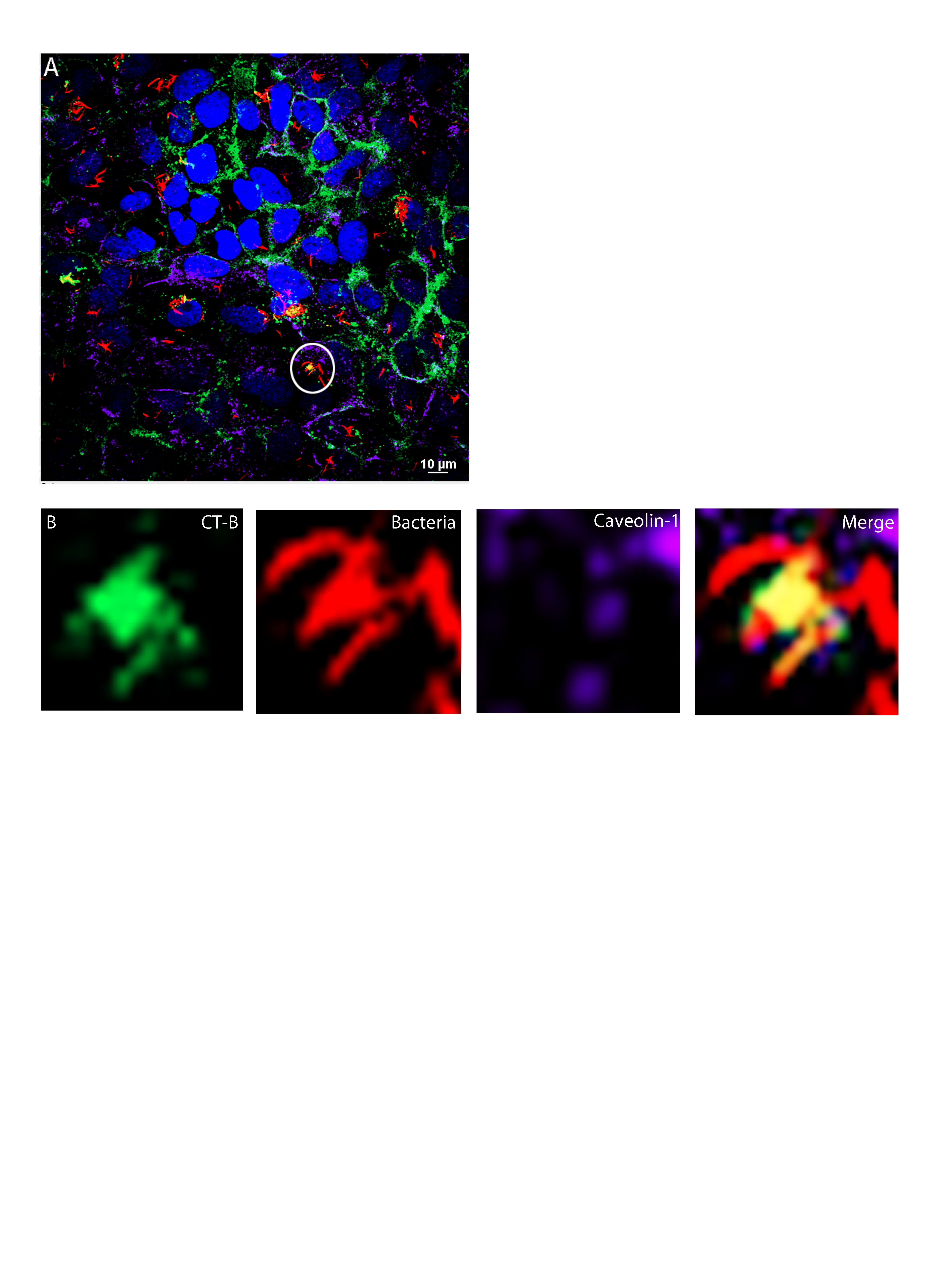

Supplement: Figure S3 — A magnified confocal image of A549 cells infected with CDC1551 demonstrates colocalization of LR markers. A549 Type II alveolar epithelial cells were infected with CDC1551 (MOI = 10) and prepared for confocal microscopy as described. Images were obtained at 63x magnification with a Nikon A1R confocal system with a Nikon Eclipse TiE confocal microscope (A). The circle indicates the area from which the magnified image was obtained. The encircled area has been cropped and magnified to illustrate the colocalization of CT-B, Caveolin-1 and bacteria for the purposes of quantification as described in the methods (B). (TIF) [file pone.0045028.s003.tif]

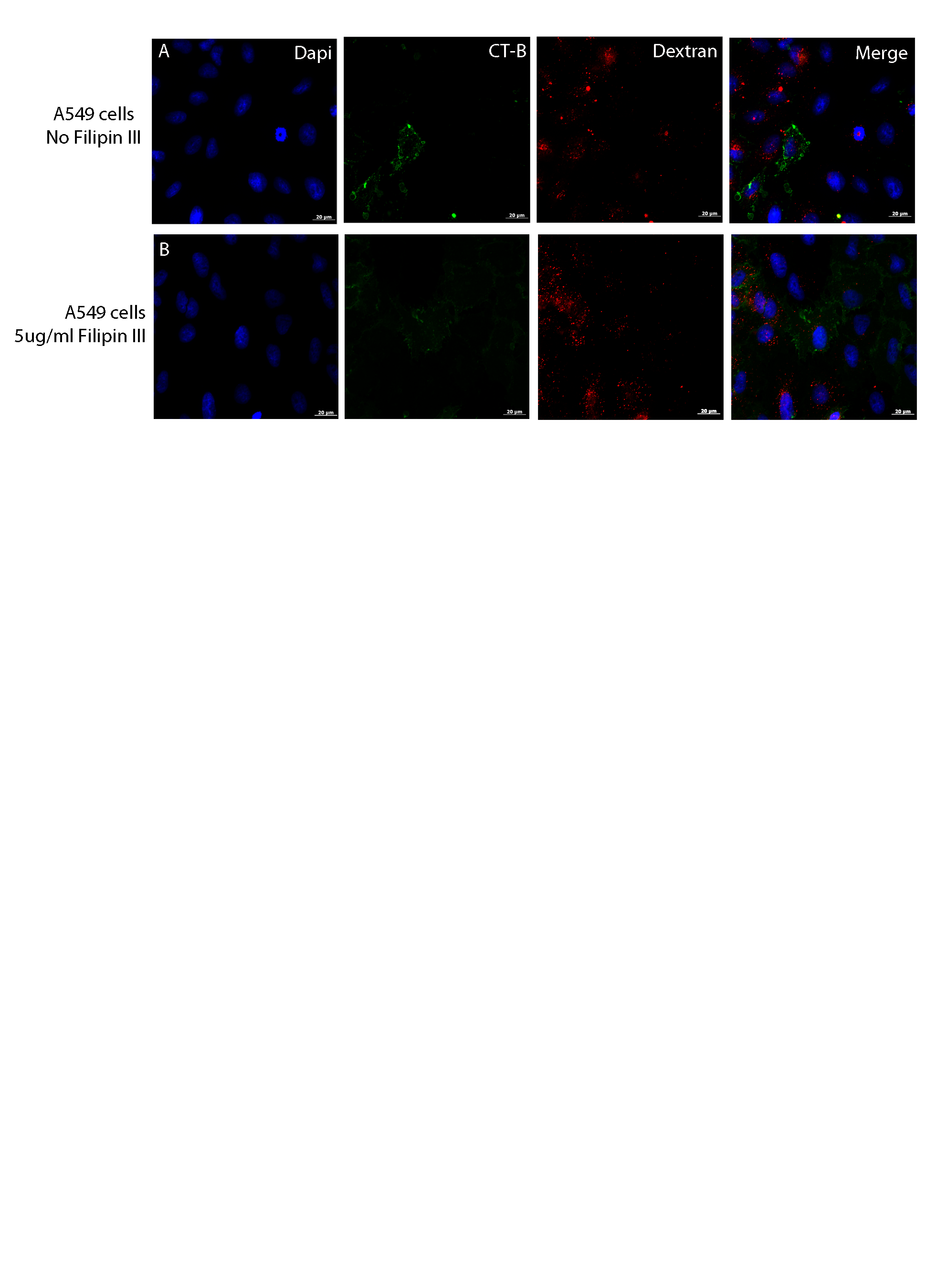

Supplement: Figure S4 — Treatment of A549 cells with Filipin III does not impact endocytosis of dextran. Filipin- (A) or non-Filipin (B) treated A549 cells were incubated with 10,000 MW dextran-TR for 30 min and uptake assessed by confocal microscopy. Quantification using ImageJ indicated no significant difference in dextran endocytosed (data not shown). Dextran treatments were performed in duplicate and repeated twice. (TIF) [file pone.0045028.s004.tif]
